# Taxonomic studies on the genus *Isotrema* (Aristolochiaceae) from China: I. *I. cangshanense*, a new species from Yunnan

**DOI:** 10.3897/phytokeys.134.37243

**Published:** 2019-10-23

**Authors:** Xin Xin Zhu, Hai Lei Zheng, Jun Wang, Yong Qian Gao, Jin Shuang Ma

**Affiliations:** 1 College of Life Sciences, Xinyang Normal University, Xinyang, Henan, 464000, China Xinyang Normal University Xinyang China; 2 Wild Dali Nature Education and Research Center, Dali, Yunnan, 671000, China Wild Dali Nature Education and Research Center Dali China; 3 Yunnan Forestry Technological College, Kunming, Yunnan, 650224, China Yunnan Forestry Technological College Kunming China; 4 Shanghai Chenshan Plant Science Research Center, Chinese Academy of Sciences, Shanghai Chenshan Botanical Garden, Shanghai 201602, China Shanghai Chenshan Plant Science Research Center, Chinese Academy of Sciences Shanghai China

**Keywords:** *
Aristolochia
*, *
Isotrema
*, morphology, subgenus *Siphisia*, taxonomy

## Abstract

*Isotrema cangshanense* X.X.Zhu, H.L.Zheng & J.S.Ma, a new species from western Yunnan, China, is described and illustrated here. It is similar to *I. utriforme*, *I. forrestianum*, *I. cucurbitoides* and *I. obliquum* The major differences between them are outlined and discussed. A detailed description, along with line drawings, photographs, habitat and distribution, as well as a comparison to morphologically similar species, is also provided. Meanwhile, the new taxon is assessed as Vulnerable (VU D2), according to the IUCN Red List criteria.

## Introduction

*Aristolochia* L. (s. l.) consists of more than 550 species (González 2012; Zhu et al. 2019a) and is the largest genus in Aristolochiaceae (Hwang et al. 2003). Three subgenera: subgenus Aristolochia, subgenus Siphisia (Duch.) Schmidt and subgenus Pararistolochia (Hutch. & Dalziel) Schmidt are recognised, based on morphological and molecular data (Wanke et al. 2006). Recently, one of the subgenera, Aristolochia subgen. Siphisia was reinstated to be an independent genus, *Isotrema* Raf., with morphological synapomorphies, such as strongly curved perianth, 3-lobed gynostemium, anthers paired on the outer surface of each gynostemium segment (Zhu et al. 2019a). In China and neighbouring countries, several species belonging to *Isotrema* have been described in recent years (Liu and Deng 2009; Xu et al. 2011; Yao 2012; Huang et al. 2013, 2015; Wu et al. 2013, 2015; Do et al. 2014, 2015a, b, c, d, 2016, 2017, 2019; Huong et al. 2014; Lu and Wang 2014; Ohi-Toma et al. 2014; Zhu et al. 2015, 2016, 2017a, b, 2018, 2019b; Gong et al. 2018; Yang et al. 2018; Li et al. 2019; Peng et al. 2019; Zhou et al. 2019). Additionally, a useful key to Asian species of *Isotrema* (Aristolochia subgenus Siphisia) is provided by Do et al. (2015a).

During an expedition to Yangbi County, western Yunnan, an unknown species of *Isotrema* was collected. Subsequent examination of herbarium specimens and study of the related literature (Hwang 1988; Ma 1989a, b; Tao 1997; Hwang et al. 2003; Do et al. 2015a; Do and Nghiem 2017; Yang et al. 2018; Zhu et al. 2019a) reveals that it is a new species described and illustrated here.

## Taxonomy

### Isotrema cangshanense

Taxon classificationPlantaePiperalesAristolochiaceae

X.X.Zhu, H.L.Zheng & J.S.Ma sp. nov.

170BBC0E-92DF-58BF-AB5C-28E83D80401E

urn:lsid:ipni.org:names:77202594-1

**Figure 1.**
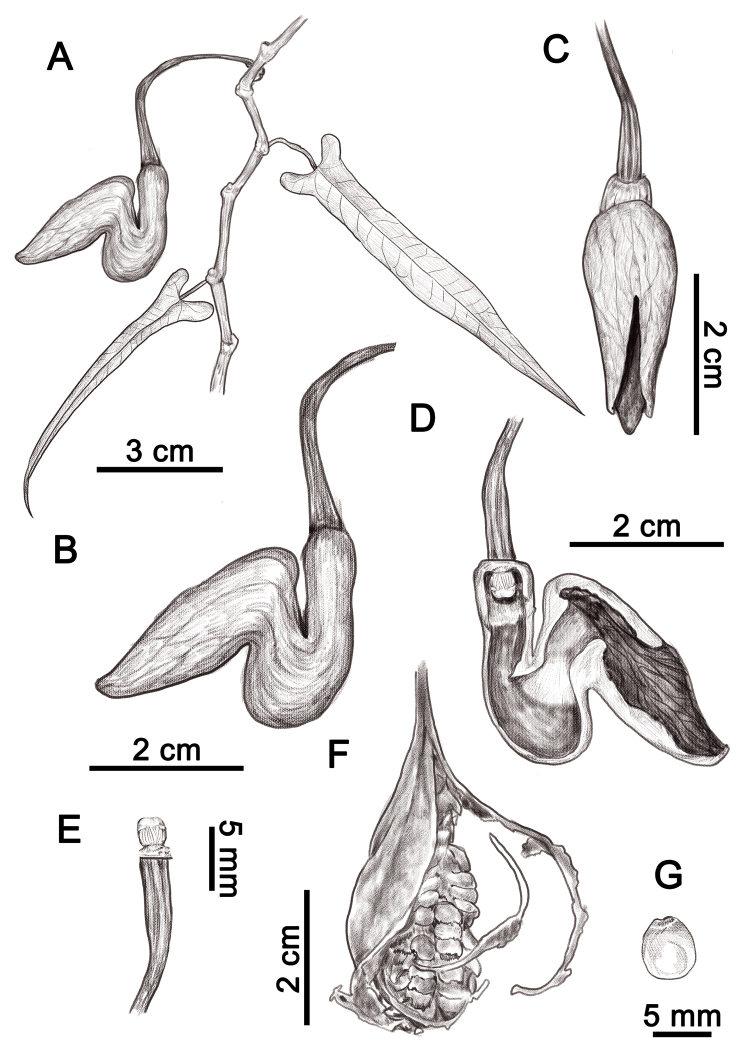
*Isotrema cangshanense* X.X.Zhu, H.L.Zheng & J.S.Ma, sp. nov. **A** habit **B** flower (lateral view) **C** flower (front view) **D** opened flower (showing the inner structure) **E** anthers and gynostemium **F** the dehiscent capsule **G** seed. Illustration by Shizhen Qiao.

**Figure 2.**
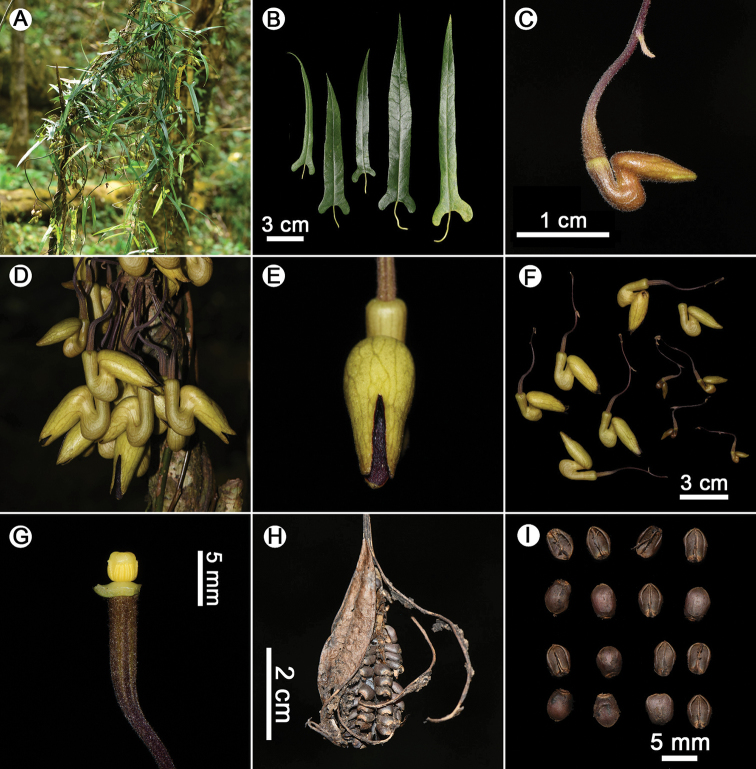
*Isotrema cangshanense* X.X.Zhu, H.L.Zheng & J.S.Ma, sp. nov. **A** habit **B** leaves **C** flower bud **D** inflorescence **E** flower (front view) **F** flowers (lateral view) **G** anthers and gynostemium **H** the dehiscent capsule **I** seeds. Photographed by Xinxin Zhu.

**Figure 3.**
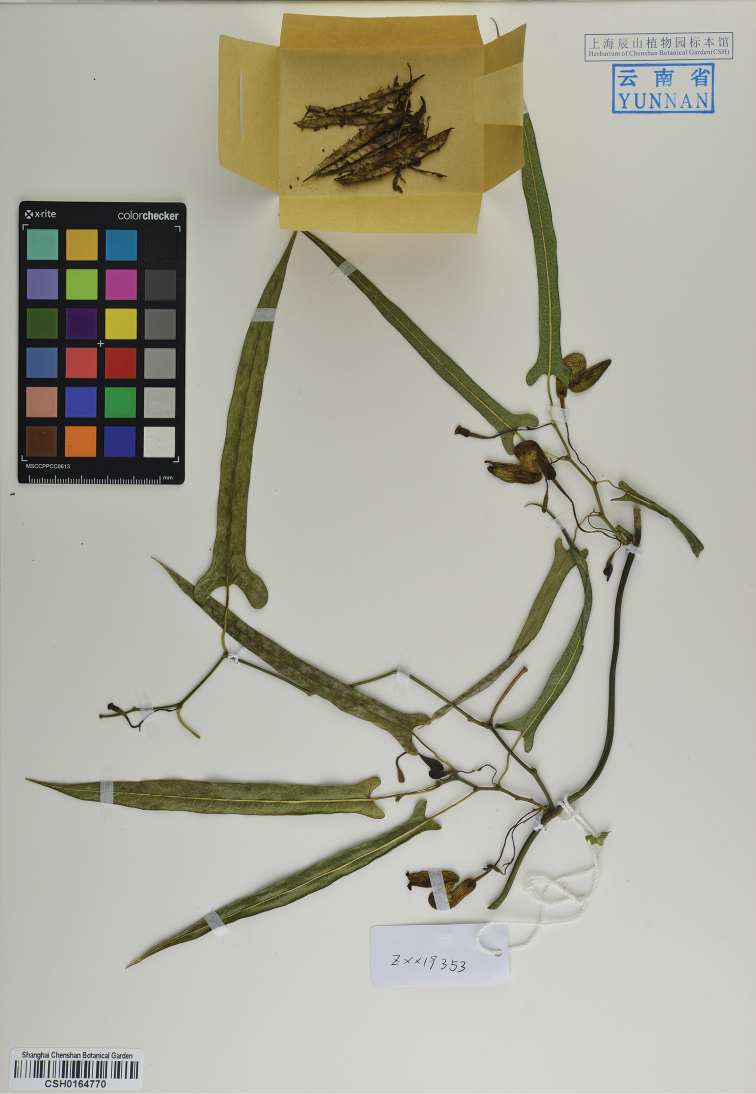
Holotype of *Isotrema cangshanense* X. X.Zhu, H.L.Zheng & J.S.Ma, sp. nov. (CSH-0164770).

**Figure 4.**
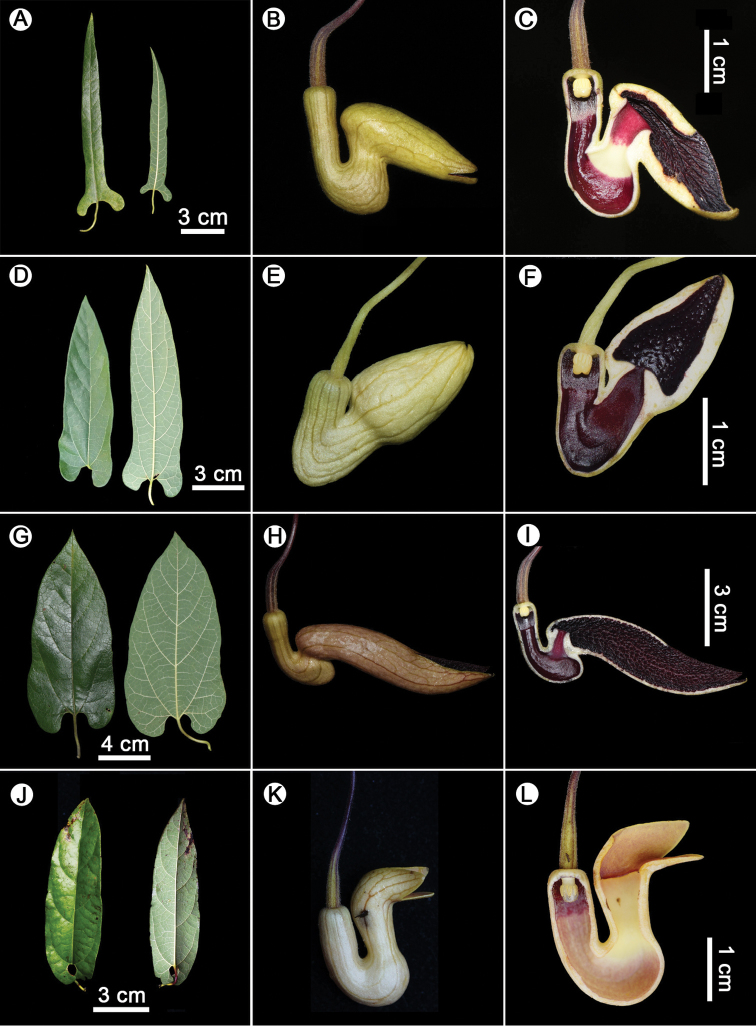
**A–C***Isotrema cangshanense* X.X.Zhu, H.L.Zheng & J.S.Ma, sp. nov. **A** leaves **B** flower (lateral view) **C** longitudinal section of flower (showing the inside structure) **D–F***Isotrema utriforme* (S. M. Hwang) X. X. Zhu, S. Liao & J. S. Ma **D** leaves **E** flower (lateral view) **F** longitudinal section of flower (showing the inside structure) **G–I***I. forrestianum* (J. S. Ma) X. X. Zhu, S. Liao & J. S. Ma **G** leaves **H** flower (lateral view) **I** longitudinal section of flower (showing the inside structure) **J–L***I. obliquum* (S. M. Hwang) X. X. Zhu, S. Liao & J. S. Ma **J** leaves **K** flower (lateral view) **L** longitudinal section of flower (showing the inside structure). **A–C, G–I** Photographed by Xinxin Zhu **D–F** photographed by Lei Cai; **J–L** photographed by Yuan Wang.

Figures 1 , 2 , 3 , 4A–C

#### Type.

China. Yunnan: Yangbi County, The Cangshan Mountain, Sancha River, 25°41'49"N, 100°02'55"E, 2239 m a.s.l., 23 April 2019, *X. X. Zhu et al. ZXX19353* (holotype: CSH [CSH-0164770!]; isotypes: CSH!, KUN!).

#### Diagnosis.

*Isotrema cangshanense* is morphologically similar to *Isotrema utriforme* (S. M. Hwang) X. X. Zhu, S. Liao & J. S. Ma, *I. forrestianum* (J. S. Ma) X. X. Zhu, S. Liao & J. S. Ma, *I. cucurbitoides* (C. F. Liang) X. X. Zhu, S. Liao & J. S. Ma and *I. obliquum* (S. M. Hwang) X. X. Zhu, S. Liao & J. S. Ma (Zhu et al. 2019a), but is distinguishable from these species by the following diagnostic characters: laminas oblong-lanceolate; calyx outside light yellow; limb narrow-ovoid, 2.4–3 × 0.9–1 cm, asymmetric, forming an acute angle with the upper part of the tube, 3-lobed, upper part separated to the middle, lower part shallowly lobed, inside black purple, net-shaped protruding stripes; throat ca. 5 mm in diam. Detailed morphological comparisons are shown in Table 1 and Figure 4.

**Table 1.** Morphological comparisons of *Isotrema cangshanense* with *I. utriforme*, *I. forrestianum*, *I. cucurbitoides* and *I. obliquum*.

Characters	*I. cangshanense*	*I. utriforme*	*I. forrestianum*	*I. cucurbitoides*	*I. obliquum*
Lamina	oblong-lanceolate, 6–20 × 1–7 cm, base auriculate, sinus 0.7–1.8 cm deep	ovate-lanceolate, 10–17 × 3–4 cm, base cordate, sinus 1–1.5 cm deep	ovate to narrowly ovate, 7–21 × 3–10.5 cm, base cordate, sinus 1.5–2 cm deep	trullate-lanceolate, ovate-lanceolate or lanceolate, 12–22 × 2.5–4.5 cm, base auriculate, sinus 1–2 cm deep	oblong-lanceolate to narrowly ovate, 12–16 × 4–6.5 cm, base cordate, sinus 1–1.5 cm deep
Inflorescence and flower	flowers in axils of leafy shoots or, on older stems, solitary or in fascicles, each fascicle with 2–6 flowers	flowers in axils of leafy hoots, solitary	flowers in axils of leafy shoots, solitary or, on older stems, solitary or in fascicles, each fascicle with 2–4 flowers	flowers in axils of leafy shoots, solitary	flowers in axils of leafy shoots, solitary
Calyx outside	light yellow	light yellow	light brown or purple	undocumented	yellowish white or pinky white
Limb shape	saccate, narrow-ovoid, asymmetric, forming acute angle with upper tube	saccate, ovoid, slightly asymmetric, straight extended from upper tube	saccate, cylinder, asymmetric, forming almost right angle with upper tube	cylindric, straight extended from upper tube	not saccate or cylindric, forming right angle with upper tube
Limb size	2.5–3 × 0.7–0.8 cm	1–2 × 0.5–1.5 cm	6–8 × 1.5–2 cm	2 × 0.2–0.3 cm	1.2–1.3 × 0.8–0.9 cm
Limb lobes	3-lobed, upper part separated to the middle, lower part shallowly lobed, inside black purple, net-shaped protruding stripes	3-lobed, shallowly lobed, inside black purple, sparse processes	3-lobed, upper part separated to middle, lower part shallowly lobed, inside black purple, densely spinous outgrowths	3-lobed, shallowly lobed, inside undocumented	3-lobed, deeply lobed, slightly asymmetric, inside light brown, smooth
Throat	ca. 5 mm in diam.	ca. 1 mm in diam.	ca. 3 mm in diam.	ca. 1 mm in diam.	ca. 6 mm in diam.

#### Description.

Woody liana. Stems terete, young shoots pubescent. Petioles 0.7–3.5 cm long, pubescent to almost glabrous; laminas oblong-lanceolate, 6–20 × 1–7 cm, adaxially almost glabrous, abaxially villous, base auriculate, sinus 0.7–1.8 cm deep, apex acute, margin entire; basal veins 2–3 pairs, palmate, 2–3 pairs from base, lateral veins 12–18 pairs, pinnate. Flowers in axils of leafy shoots or, on older stems, solitary or in fascicles, each fascicle with 2–6 flowers; pedicels 1.5–4 cm, pubescent partly villous; bracteole 1, lanceolate, 2–5 × 1–2 mm, adaxially glabrous, abaxially densely villous, inserted below the middle of pedicel. Calyx tube geniculately curved, outside light yellow, abaxially sparsely pubescent partly villous; basal tube 1.8–2.5 × 0.5–0.6 cm, inside black purple, densely villous at base; upper tube 1.3–1.6 × 0.6–0.8 cm, inside black purple at base, light yellow towards apex, light red at upper part; limb saccate, narrow-ovoid, asymmetric, forming acute angle with upper part of the tube, 2.5–3 × 0.7–0.8 cm, 3-lobed, upper part separated to the middle, lower part shallowly lobed, upper two lobes triangular-lanceolate, 1.2–1.5 × 0.4–0.5 cm, lower lobe triangle, 0.4–0.7 × 0.4–0.5 cm, inside black purple, net-shaped protruding stripes; throat ca. 5 mm in diam. Anthers 6, oblong, ca. 1.5 mm long, adnate in 3 pairs to base of gynostemium, opposite to lobes. Gynostemium ca. 2.7 × 2.5 mm, 3-lobed. Ovary terete, ca. 10 mm long, densely villous. Capsule obovate-elliptic, ca. 4.5 × 2 cm. Seeds obovate-elliptic, ca. 5 × 4 mm, not winged, the adaxial surface deeply concave and the abaxial surface convex, both surfaces glabrous.

#### Phenology.

Flowering from April to May, fruiting is predicted from July to August (we have seen is just two residual fruit from last year).

#### Etymology.

The specific epithet derives from the type locality, The Cangshan Mountain, Yangbi County, western Yunnan, south-western China. The Chinese name is given as “苍山关木通”.

#### Distribution and habitat.

The new species is currently known only from the Cangshan Mountain, Yangbi County, Yunnan, China. It grows in forests at an elevation between 2239 m and 2379 m, together with *Castanopsis* sp. (Fagaceae), *Disporum* sp. (Colchicaceae), *Notochaete hamosa* Benth. (Lamiaceae), *Photinia* Lindl. (Rosaceae), *Rubus* sp. (Rosaceae) etc.

#### IUCN Red List category.

Since *Isotrema cangshanense* is known from one population only, with fewer than ten individuals, the new species is assigned a preliminary status of Vulnerable (VU D2) according to IUCN Red List Criteria (IUCN 2012), indicating a population with a very restricted area of occupancy (typically less than 20 km^2^) or number of locations (typically five or fewer). Although the area is under protection as a national nature reserve, habitat disturbance brought about by human activities, such as grazing and felling, may have a negative impact on the new species.

#### Specimens examined (Paratypes).

**China. Yunnan**: Yangbi County, The Cangshan Mountain, 2300 m a.s.l., 23 April 2019, *X. X. Zhu et al. ZXX19354* (CSH!); the same location, 2379 m a.s.l., 23 April 2019, *X. X. Zhu et al. ZXX19355* (CSH!).

## Discussion

*Isotrema cangshanense* has a horseshoe-shaped perianth, a 3-lobed gynostemium, each lobe fused with one pair of oblong stamens which are characteristics for the genus *Isotrema* (Zhu et al. 2019a). The new discovery, along with many new species recently discovered from China (Liu and Deng 2009; Xu et al. 2011; Huang et al. 2013, 2015; Wu et al. 2013, 2015; Lu and Wang 2014; Do et al. 2015a; Zhu et al. 2015, 2016, 2017a, b, 2018, 2019b; Gong et al. 2018; Yang et al. 2018; Li et al. 2019; Peng et al. 2019; Zhou et al. 2019), provide evidence that the genus *Isotrema* is very diverse in China. Moreover, referring to Ohi-Toma et al. (2014), Do et al. (2015a), Li et al. (2019), Peng et al. (2019), Zhou et al. (2019) and Zhu et al. (2019a, b), there are 64 species and one subspecies of *Isotrema* in China. It is predicted that more new species of *Isotrema* will be discovered when more field investigations are conducted in this region.

## Supplementary Material

XML Treatment for Isotrema cangshanense
